# Supplementary material for: Combined Inhibition of FOSL-1 and YAP Using siRNA-Lipoplexes Reduces the Growth of Pancreatic Tumor
Source: Cancers (Basel). 2022 Jun 24;14(13):3102. doi: 10.3390/cancers14133102 (PMC9265026; doi:10.3390/cancers14133102)
Supplement: Supplementary file 1 [file cancers-14-03102-s001.zip › cancers-1753204-supplementary.pdf]

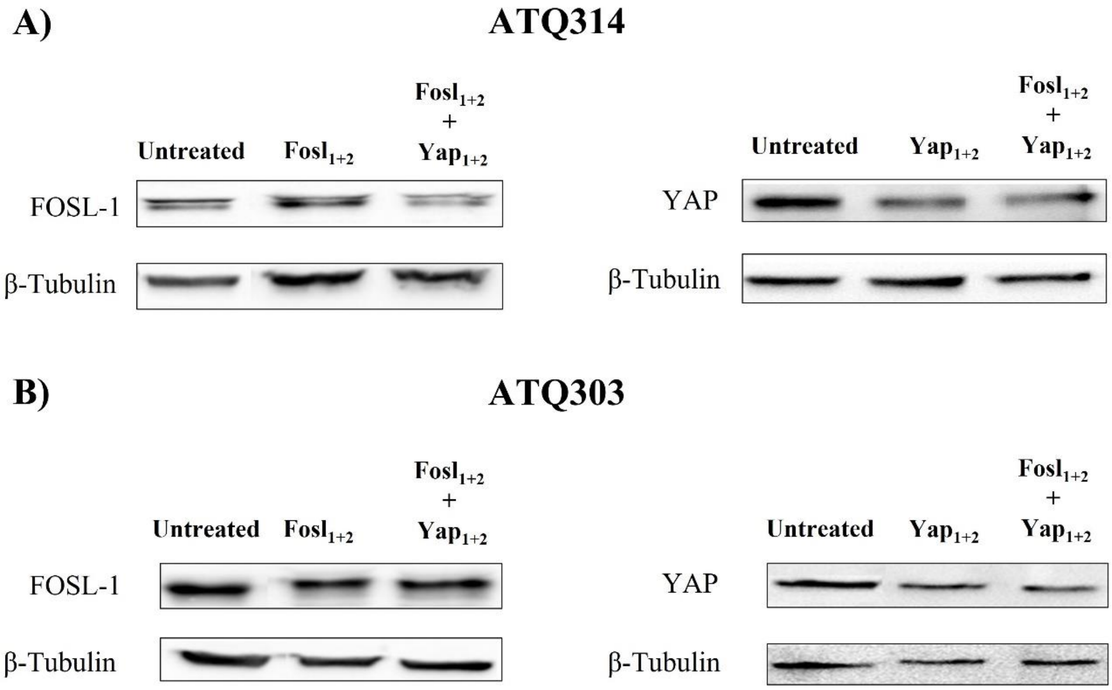

Figure S1. Representative Western blot of in vitro silencing efficacy of lipoplexes in ATQ314G (A) and ATQ303G (B) after 72 h of incubation with the different treatments (lipoplexes charge ratio (±) 15 at siRNA final concentration of 50 nM). The images has been cut to facilitate the comparison between treatments and cell lines. The original blots are shown in Figure S2.

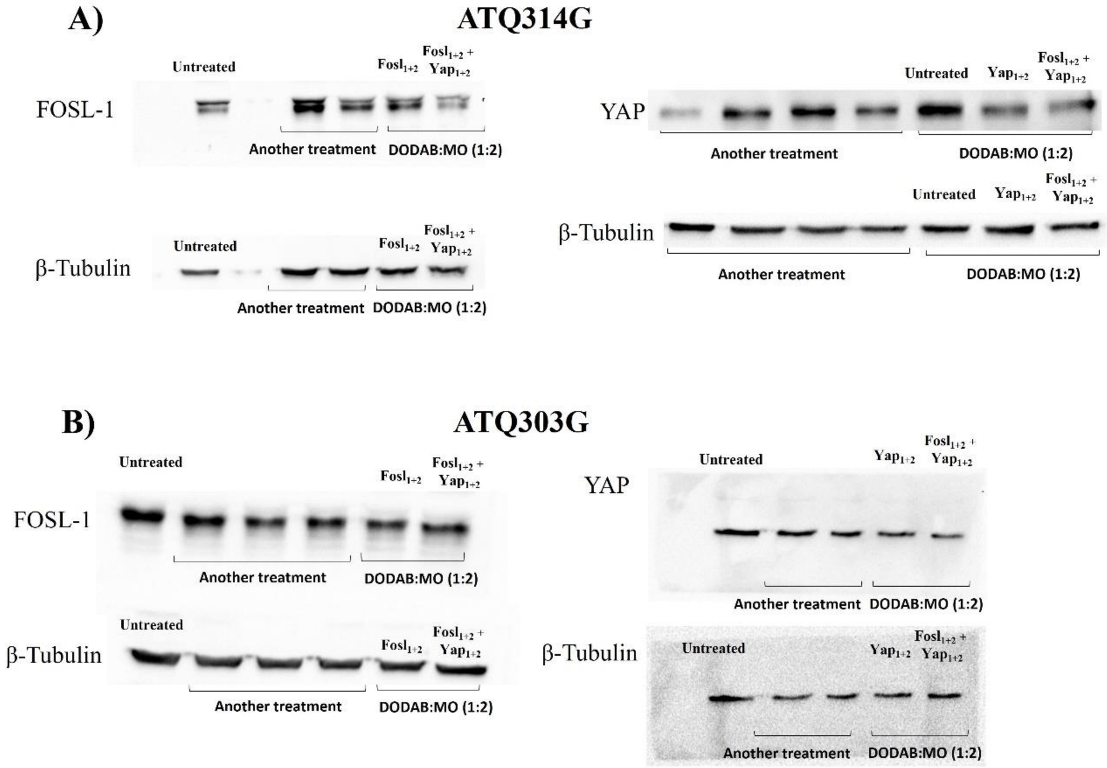

Figure S2. Original Western blot images of in vitro silencing efficacy of lipoplexes in ATQ314G (A) and ATQ303G (B).

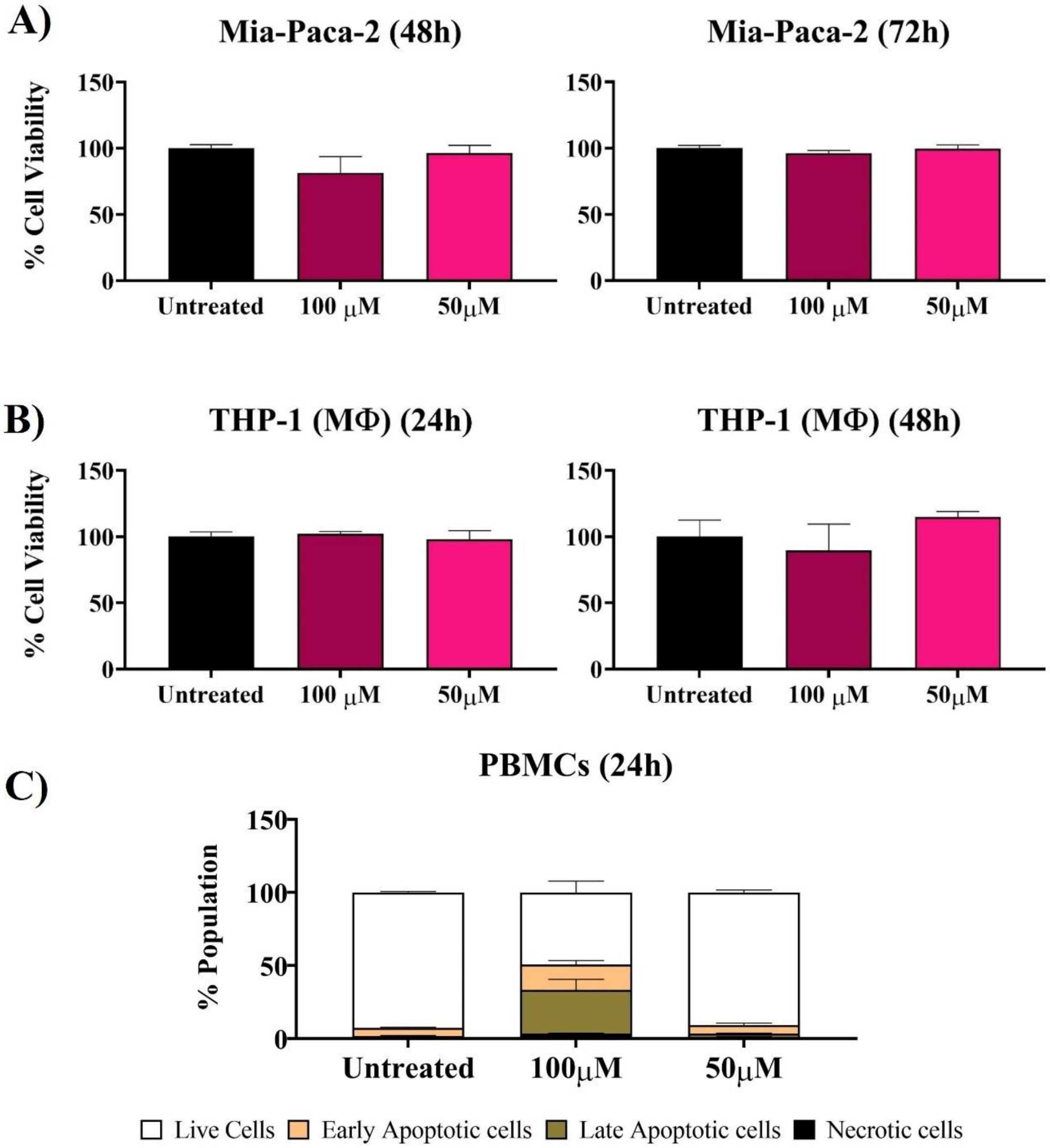

Figure S3. Viability of different cell lines in the presence of liposomes. A) Percentage of cell viability of MIA-PACA-2 at 48 and 72 h determined by MTS assay. B) Percentage of cell viability of macrophage-differentiated THP-1 at 24 and 48 h determined by MTS assay. **C)** Percentage of different cell populations (live, early apoptotic, late apoptotic and necrotic cells) of peripheral blood mononuclear cell (PBMCs) after 24 h determined by flow cytometry.

Hemocompatibility of the liposomes and lipoplexes

The interaction with blood components was also studied. The complement system plays an important role in the innate response and also in the homeostasis [48]. In the last years, it has been reported that complement system can have both a pro- as well as an anti-tumour role, depending on the type of cancer [49]. Specifically, in mice and patients with pancreatic tumours, it has been observed an increase in the expression of complement genes, like C1QA or C1QB [50]. In general, the release of complement factors (anaphylatoxins C5a and C3a) is found in the tumour microenvironment, which favors the proliferation, angiogenesis, and metastasis [51,52]. However, the real effect or influence of the complement activation in pancreatic cancer is still uncertain [53]. DODAB:MO (1:2) liposomes induced a significant activation of the complement system at the highest concentrations tested (100 and 50 μM), measured by the degradation of the C3 factor by Western blot (**Figure S4A**). However, at low concentration (25 μM) and when the siRNA is

encapsulated and lipoplexes are formed, the activation decreases to basal levels. The fact that DODAB:MO (1:2) liposomes do not induce complement activation at low concentrations and, above all, as a siRNA nanocarriers, is very beneficial, because the pro-inflammatory environment will not be favoured if used in therapy.

Likewise, the hematocompatibility is a fundamental aspect for its use in medicine because liposomes could interact with erythrocytes after entering into the body [41]. If they produce a breakdown of the erythrocytes, they would automatically be discarded as therapeutic drug to avoid possible multi-organ failure. Only at the highest concentration (100 μM) tested, liposomes were partially haemolytic (2-5%, according to the ASTM International protocol E2524-08, see material and methods) (**Figure S4B**). As observed with complement activation, lipoplexes reduce the partial haemolytic effect of liposomes.

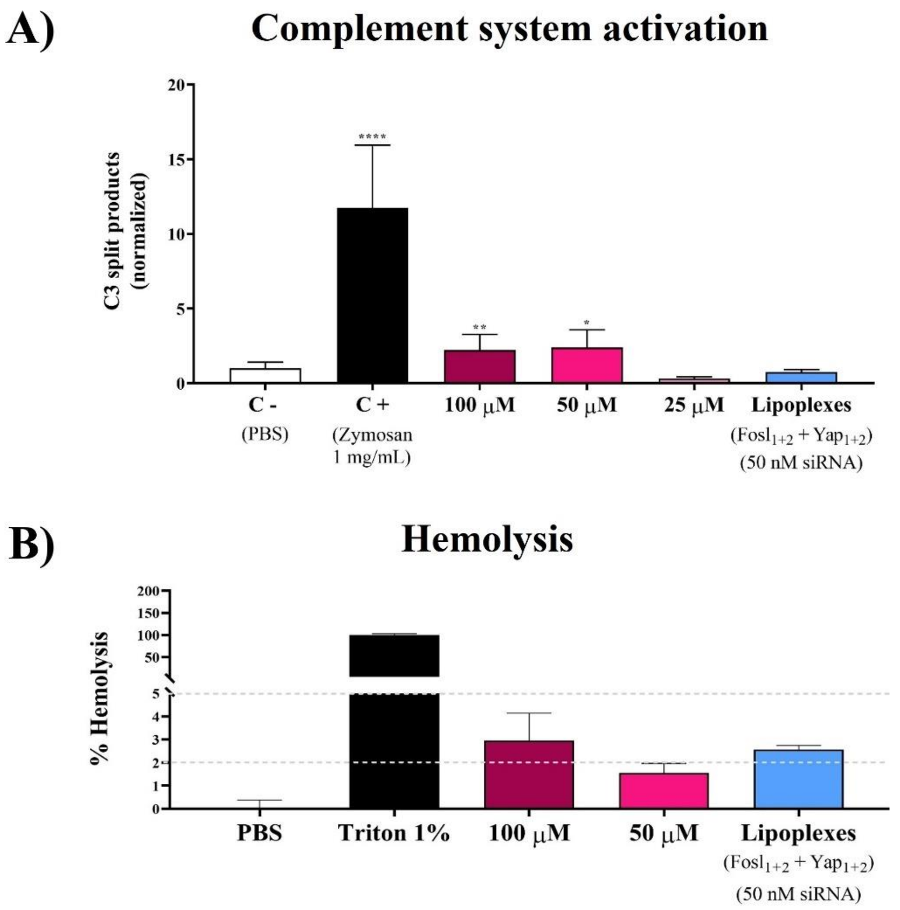

Figure S4. Hemocompatibility of liposomes and lipoplexes. A) Quantification of C3 factor degradation induced by liposomes at 100, 50 and 25μM and lipoplexes at 100 μM (containing 50 nM siRNA) determined by Western blot. **B)** Percentage of hemolysis induced by liposomes at 100 and 50 μM and lipoplexes at 100 μM (50 nM siRNA). Lipoplexes $Fosl_{1+2}$ + Yap_{1+2} were selected as an example. Zymosan at 1 mg/mL and 1 % Triton, were used as positive control for complement activation and hemolysis, respectively, and PBS as negative control for both assays. In the graphs, the statistically significant differences between PBS and the treatments were represented as: * $p \leq 0.05$, ** $p \leq 0.01$. **** $p \leq 0.0001$.
